# Operational Challenges of an Asia-Pacific Academic Oncology Clinical Trial

**DOI:** 10.1200/GO.23.00040

**Published:** 2023-06-26

**Authors:** Daphne Day, Han Chong Toh, Raghib Ali, Estelle Mei Jye Foo, John Simes, John Whay Kuang Chia, Eva Segelov

**Affiliations:** ^1^ Faculty of Medicine, Nursing and Health Sciences, Monash University, Melbourne, VIC, Australia; ^2^ Department of Oncology, Monash Health, Melbourne, VIC, Australia; ^3^ Division of Medical Oncology, National Cancer Centre Singapore, Singapore; ^4^ Public Health Research Centre, New York University, Abu Dhabi, United Arab Emirates; ^5^ University of Sydney NHMRC Clinical Trials Centre, Sydney, NSW, Australia; ^6^ Faculty of Medicine, University of Bern, Bern, Switzerland

## Abstract

**PURPOSE:**

The Asia-Pacific (APAC) region is a major focus for multinational clinical trials, although its cultural, linguistic, economic, and regulatory diversity pose significant challenges for trial conduct, particularly for academic clinical trials.

**METHODS:**

We describe our experience running the investigator-initiated phase III randomized, fully accrued, Aspirin for Dukes C and high-risk Dukes B Colorectal cancer trial (ASCOLT, ClinicalTrials.gov identifier: NCT00565708, N = 1,587), studying the benefit of aspirin in resected high-risk colorectal cancer. ASCOLT opened in 2008 and is the first large academic adjuvant trial fully conducted in the APAC region. Centrally coordinated by the Trial Management Team at the National Cancer Centre Singapore, it has involved 74 sites across 12 APAC countries/regions, including five middle-income countries.

**RESULTS:**

Challenges encountered included regulatory complexity, communication and logistical barriers, limited funding and resources, disparate experience and infrastructure across sites, recruitment holds because of changes in local laws, patient attrition, and disruptions caused by the COVID-19 pandemic. Over 100 contracts and 49 ethics board reviews were required, contributing to a lengthy prestudy preparation time of 2 years and start-up times of approximately 6 months per site. Some of the mitigating actions included engaging local cooperative groups (eg, the Australasian Gastro-Intestinal Trials Group in Australia and New Zealand) and seven contract research organizations to manage sites, regular communication with the central team, transition to electronic data management, and a centralized drug-dispensing system.

**CONCLUSION:**

To ensure an efficient and patient-centered clinical trials environment in the APAC region and sustained growth, we suggest coordinated approaches to harmonize regulatory processes, APAC academic oncology trials consortia to streamline processes and provide governance, and ongoing commitment from governments, funding agents, and industry.

## INTRODUCTION

Multinational clinical trials can expedite recruitment, enable diverse geographic representation, fulfill licensing requirements by multiple regulators, and have been pivotal in improving cancer care outcomes. The past decade has seen exponential growth in clinical research in the Asia-Pacific (APAC) region coupled with economic advances, increasing health care expenditure, and government investment in research and development. However, coordinating clinical trials across international borders in a region rich with cultural, linguistic, and economic diversity is extremely complex. These challenges are amplified for investigator-initiated trials, which are often geared toward answering clinical practice–related but less commercially driven questions, and typically lack the financial and specialized human resources of industry-sponsored trials. In a global analysis of over 119,000 registered clinical trials between 2006 and 2013, 80.1% of international trials were industry-funded.^1^ In fact, only 3.2% of non–industry-sponsored trials were international compared with 30.3% of industry-sponsored trials.^1^ Several survey-based studies of investigators from various medical disciplines in North America and Europe have identified the primary barriers hindering the conduct of investigator-initiated studies as lack of funding and time, and delays caused by regulatory and administrative processes.^2-4^

**CONTEXT**

**Key Objective**
To highlight the challenges of running a large phase III academic oncology multinational clinical trial (Aspirin for Dukes C and high-risk Dukes B Colorectal cancer trial [ASCOLT]), investigating the benefit of aspirin in high-risk curatively treated colorectal cancer across 12 Asia-Pacific (APAC) countries/regions.
**Knowledge Generated**
Key challenges included complex regulatory requirements, limited funding, disparities in infrastructure across sites, and recruitment and operational delays. Solutions included a simple and pragmatic trial design, obtaining continual academic and philanthropic funding, engaging local cooperative groups and contract research organizations, developing processes sensitive to local needs with continual adaptation, and centralized electronic data management and drug-dispensing system.
**Relevance**
While ASCOLT highlights the feasibility and value of APAC academic collaborative trials, our challenges reflect the complexities in a region of cultural, linguistic, economic, and regulatory diversity. Regulatory harmonization, government and industry investment, a patient-centered approach, and an APAC academic trials consortium are suggested.

**FIG 1:**
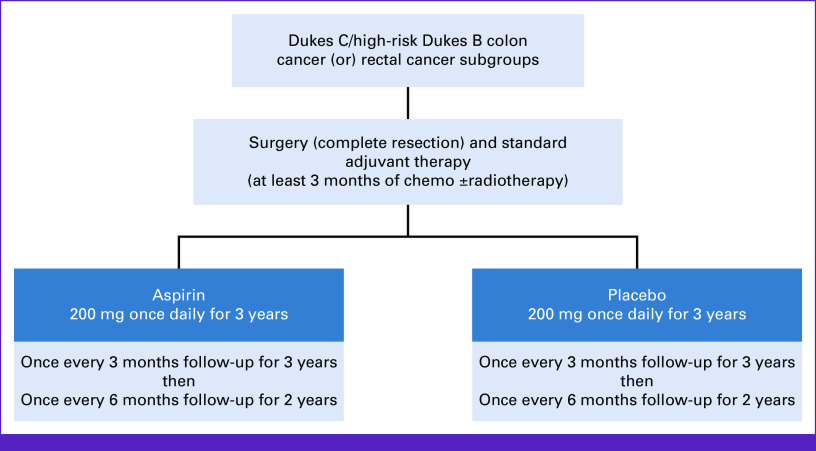
ASCOLT trial schema. ASCOLT, Aspirin for Dukes C and high-risk Dukes B Colorectal cancer trial.

**FIG 2:**
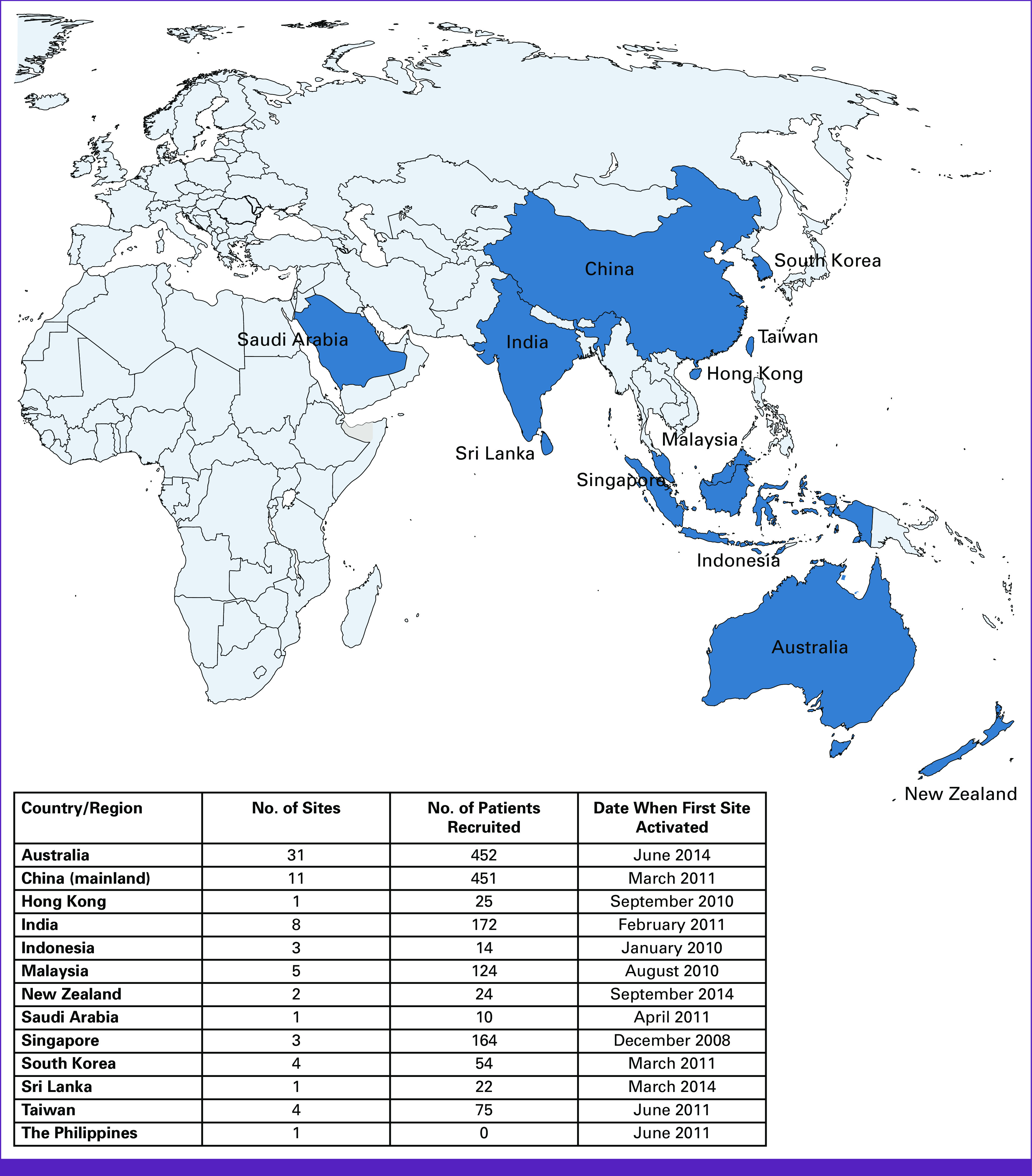
Map of participating countries and/or regions.

Herein, we describe our experience running the international investigator-initiated phase III randomized, placebo-controlled, Aspirin for Dukes C and high-risk Dukes B Colorectal cancer trial (ASCOLT, ClinicalTrials.gov identifier: NCT00565708),^5^ highlighting the challenges encountered, particularly, limited resources and regulatory complexity. The trial investigates the benefit and safety of aspirin 200 mg once daily for 3 years in patients with high-risk stage II and III colorectal cancer (CRC) after standard curative treatment (surgical resection and adjuvant systemic therapy; Fig 1). ASCOLT opened in 2008 and is the first APAC-run large academic adjuvant trial, involving 74 centers across 12 countries and/or regions, including five middle-income countries (Fig 2).^6^ The trial has fully accrued (N = 1,587) and is expected to report results in third quarter of 2023.

## ASCOLT OVERVIEW: CONCEPTION AND CORE INFRASTRUCTURE

CRC is the third most common cancer globally and the second most common cause of cancer deaths, with increasing incidence in Asian countries such as China, Japan, India, and Singapore, as well as middle- and low-income countries, likely related to greater uptake of Western lifestyles.^7^ Approximately 80% of patients with CRC present with early-stage disease, although 25%-50% may recur after curative therapy, usually within the first 5 years.^8-10^ The study premise was founded on growing evidence from a large body of epidemiologic and observational data supporting a role of aspirin in CRC primary and secondary prevention and approximately 30%-50% reduction in CRC-specific mortality.^11-15^ The primary end point is disease-free survival and the secondary end points are 5-year overall survival and survival outcomes in subpopulations of interest.

ASCOLT was purposefully designed to run entirely in the APAC region. If positive (either for all comers or a biomarker-selected population), the trial will have a major practice-changing impact in both the APAC and globally, as aspirin is widely available, cheap, relatively safe, and familiar to both patients and clinicians. There are seven other phase III adjuvant aspirin CRC studies currently running internationally (Table 1), reflecting the level of interest and potential public health benefit in repurposing aspirin; ASCOLT is expected to be the first to report.^16,17^ Six of these trials and ASCOLT have an agreement to share experience and data as part of the Aspirin Trialist Collaborative Group.^18^

**TABLE 1:**
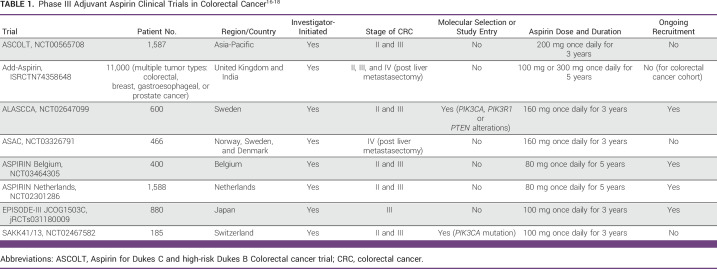
Phase III Adjuvant Aspirin Clinical Trials in Colorectal Cancer^16-18^

ASCOLT is centrally coordinated by the Trial Management Team at the National Cancer Centre Singapore (NCCS). The team consists of the co-chief primary investigators (J.C. and H.T.) and two full-time staff including a project manager (E.F.) and an assistant program manager. Data management and statistical services are provided by the Singapore Clinical Research Institute (SCRI). The four study chairs include J.C. and H.T. and two non–Singapore-based co-chairs (R.A. and E.S.). The trial steering committee (10 members, including two independent of the trial and one observer) provides study oversight and meet annually with additional ad hoc meetings. The International Data Monitoring Committee comprising three experts met at the two interim analysis points to independently review safety and efficacy data. A translational subcommittee (chair, E.S.) was formed in 2014 to manage biospecimen collection and correlative studies.

Initial funding was obtained from Singhealth in 2007 and the National Medical Research Council, Singapore, in 2009. Grant funding was also obtained from the National Health and Medical Research Council for Australian and New Zealand participation in 2014 and for translational research in 2016.

To develop and maintain processes sensitive to country-wide and local site needs, particularly language and regulatory, the central team contracted local cooperative groups and seven clinical research organizations (CROs). For example, in China, a CRO provides the operational support and monitoring for all sites and facilitates communication with the Singapore team. The Australasian Gastro-Intestinal Trial Groups (AGITG) sponsors the trial at the 31 Australian and New Zealand sites. In other countries, each site or health group acts as its own sponsor.

**FIG 3:**
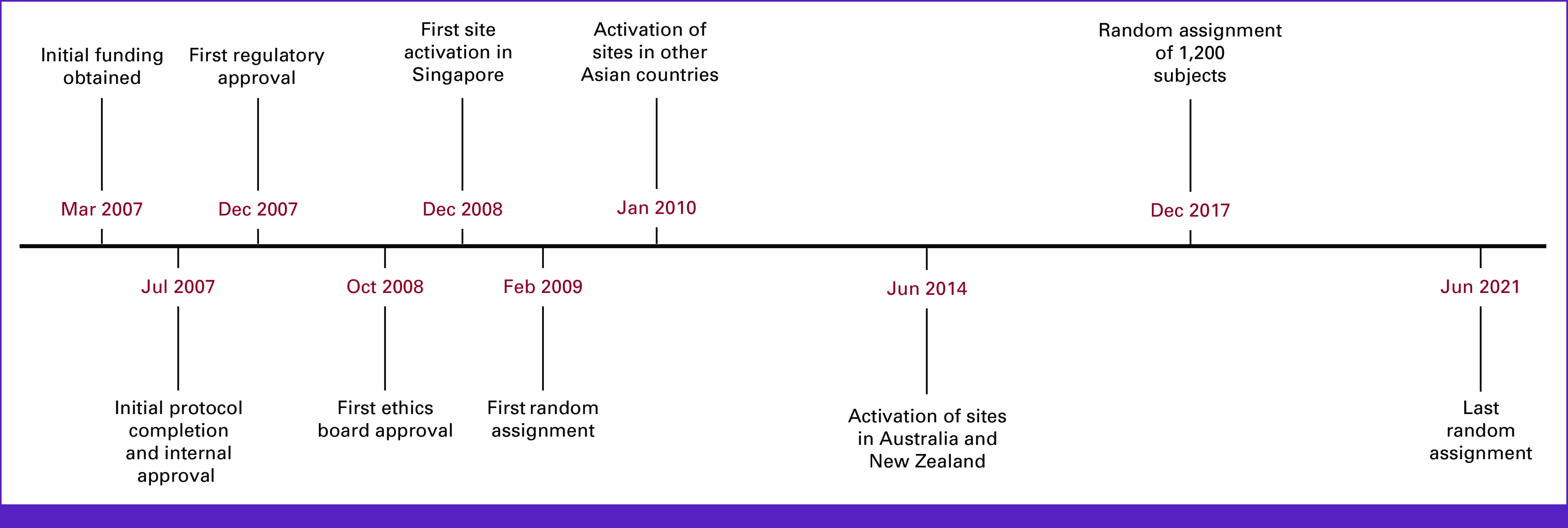
Timeline of key trial milestones.

The study first opened in Singapore in December 2008, followed by other Asian sites, and Australia and New Zealand in 2014 (Fig 3). Recruitment was completed in June 2021 and database lock is scheduled for May 2023. The total number of protocol versions was seven.

## CHALLENGES ENCOUNTERED DURING TRIAL CONDUCT

We encountered the common logistic challenges of multicentered trials, such as inconsistent compliance with study procedures across sites, high staff turnover, and scheduling of meetings across time zones. Aspects of particular relevance to the APAC region are discussed below (Table 2).

**TABLE 2:**
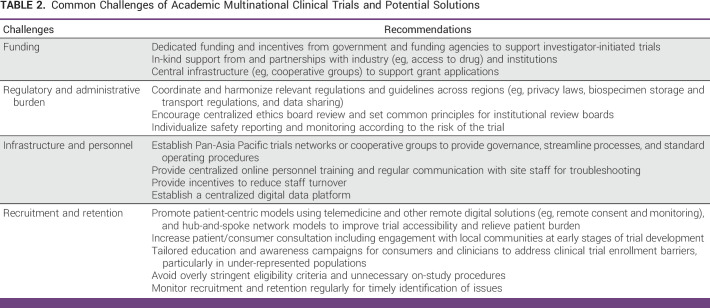
Common Challenges of Academic Multinational Clinical Trials and Potential Solutions

### Regulatory, Contractual, and Ethics Requirements

As each of the 12 countries and/or regions has its own regulatory system and multiple organizations and stakeholders were involved, contractual and administrative preparations were complex and laborious, with frequent delays. In total, over 100 contracts and 49 ethics board reviews were required, contributing to the protracted 2-year prestudy preparation time and start-up times of approximately 6 months per site. In addition to multiplicity of reviews, ethics boards can also vary substantially in their application procedures, level of scrutiny during reviews, and interpretation of regulations and scientific evidence,^19^ as was our experience. For indemnity arrangements, the Singapore Ministry for Health assigned an insurance company that brokered local agreements and policies. Where local trial insurance was not available or not sought, a master insurance policy provided indemnity coverage (eg, in Sri Lanka and Hong Kong).

Ethical, cultural, and legal considerations relating to biological specimen handling are particularly diverse across regions, requiring bespoke strategies. Biospecimen collection was limited to sites with adequate capacity and permissive regulations (centers in China, Australia, Malaysia, New Zealand, Singapore, and Taiwan), with a dedicated Biospecimen Handling Manual written to enable consistent processes. Countries including China have restrictions against the outward transport of biospecimens. Parallel collection and analysis procedures were thus set up in China. For Singapore, laws on sample export changed during the study period, again necessitating complex contracts between countries and academic centers. Moreover, in a lengthy trial, modifications in regulations at the national and/or local levels were not infrequent, with some significantly affecting trial timelines. For example, a regulatory change in China halted recruitment and data sharing across all Chinese sites for 18 months, which delayed data transfer and recruitment completion by approximately 1 year.

### Operational, Data Management, and Drug Supply Aspects

Consent forms and case report forms were translated into 13 languages. Criteria for site selection included existing collaborations, site experience, and resources. The local CRO or cooperative group performed personnel training, including site initiation visits and on-site or remote monitoring. Protocol training was undertaken by the central team to standardize interpretation and implementation. The central team communicated monthly with local CROs for status checks and troubleshooting. No regular investigator meetings occurred because of limited funding.

An overarching collaboration agreement allowed data sharing among the Asian sites. In China, legal requirements permitted data sharing between sites and the Singapore team only. A separate data sharing agreement was set up for Australia and New Zealand. Data management transitioned to electronic medical records early in the trial in 2010, which relieved paper-system inefficiencies, and enabled better maintenance of data uniformity and real-time queries. Data entered via case report forms by site staff were transferred centrally and stored at SCRI. At Asian sites, the local CRO performed on-site data verification and in Australia and New Zealand, regular data quality audits were conducted. Centrally, on average, over 200 data queries are raised monthly by the SCRI data management team. Given varying experience and data quality across sites, an additional step is performed by the Project Managers by way of monthly data logic checks and review of submitted serious adverse events.

Drugs were sponsored by Bayer and a Singapore-based depot was set up from 2012 for drug storage and distribution, and destruction of expired drug. Random assignment to aspirin or placebo was based on two predetermined stratification factors via a central randomization web system. Drugs were labeled using statistician-stratified kit numbers before distribution to sites. The central project management team monitors the dispensing rates at each site and estimates ongoing supply quantity to minimize waste and ensure that local storage capacities are not exceeded. A streamlined supply chain has been less susceptible to errors and extraneous circumstances, and circumvents the issue of a lack of pharmacy staff at many sites. An additional depot and intermediary step were necessary in China because of its importation conditions, and this was also coordinated by the Singapore team.

As is commonly encountered in large clinical trials, trial milestones were often behind initial forecasts (Fig 3). First, we observed slower accrual than original projections. Site numbers were increased from 10 in 2010 and steadily to 74 in 2017. As more sites were initiated, the recruitment rate increased to approximate the target rate from 2013. A second issue was lower-than-expected event numbers, a frequent phenomenon of modern adjuvant oncology clinical trials, in the setting of more precise staging and therapeutic improvements. On the basis of an interim data analysis of pooled event rates in 2018, the Steering Committee revised the enrollment target (from 1,200 to 1,587) and period (extended by 5 years). Third, operational delays and interruptions occurred at the overall study, country-specific, and site-specific levels; the COVID-19 pandemic further hampered study progress.

### Resource Constraints and Disparities

Extended timelines significantly increased running costs, necessitating judicious planning of resource acquisition and allocation. Although ASCOLT was initially government-funded, philanthropic funding became the main source over the past 5 years and currently comprise over 90% of total funding sources. It is noteworthy that at NCCS and in Singapore, a pre-existing infrastructure such as a clinical research center was not in place and processes were not fit for purpose for trial coordination and management. This imposed considerable workload on the core team who had to fulfill numerous roles and adapt over time, including repeated efforts to secure competitive grants; all four study co-chairs are full-time clinician-researchers with multiple commitments. Recognizing the relatively low-risk nature of the trial, given the well-known safety profile of aspirin, the study was designed to be pragmatic and simple, with rationalized data collection focusing on relevant end points to further reduce cost and redundancies. Moreover, sites followed local surveillance schedules for computed tomography scans and colonoscopy aside from a few minimum requirements, to intentionally reflect real-world practices. Substudies including correlative work were set up separately and as funding (largely independent of the main study) became available.

Whereas industry-funded trials can provide a per-participant budget to offset treatment and visit costs, academically run trials tend to have much smaller budgets. The ASCOLT budget covered core trial infrastructure only with very limited contribution to patient-related costs, which relied on in-kind provision by sites and in some cases supplemented by local academic funding (eg, in Australia and New Zealand). Not surprisingly, resource shortfalls, including institutional support for participant visits and follow-up and ancillary procedures disproportionately affected countries where health care is not publicly funded.

### Participant Enrollment and Retention Challenges

Recruitment rates varied widely across countries and sites and some of the challenges were likely context and location dependent. At the participant level, factors such as socioeconomic disadvantage, cultural perceptions, health literacy, and the absence of universal health care may render clinical research less appealing to the target population. In relatively resource-poor areas, these factors may play a more prominent role. However, clinical trial participation may be an avenue to access health care. In countries and centers with more active clinical research, alternative trials including those of novel agents may have competed for the same patient population. Naturally, the number of participating centers per country and the population size served also affected recruitment.

At the site level, resource disparities can have a myriad of downstream effects such as infrastructure support, staff availability, and experience, all of which can directly affect recruitment, as well as trial adherence and follow-up. In fact, of the 74 activated sites, only 66 recruited patients. Seven sites were closed because of resource or recruitment issues. Australia (n = 452) and China (n = 451) contributed the highest number of participants, despite the former joining at least 4 years after other countries. Australian sites also collected the bulk of the biospecimens with available baseline tumor samples and serial blood specimens for approximately 90% and 80% of participants, respectively. The relative success in Australia is likely because of a combination of factors including high number of sites (n = 31), a reliable public health system, established clinical trial infrastructure including biospecimen collection, additional continual academic funding and importantly, a well-established national cooperative trials group (AGITG) acting as a sponsor, providing overall coordination and study oversight.

Medication adherence was monitored by site coordinators using accountability logs and verified by the local CROs, with 89.8% compliance rate observed at the last analysis in February 2022. Phone reminders were used to prompt participants about upcoming visits. Premature treatment discontinuation and patient attrition because of reasons other than disease recurrence or significant toxicity was identified as a key issue, and disproportionately affected certain sites and regions. Local protocol-specific training and improved communication with the central team partly alleviated this issue. The February 2022 analysis found that 12.7% of participants had discontinued early for reasons other than recurrence, death or toxicity, or were lost to follow-up. If confirmed at the final analysis, higher-than-anticipated attrition rate (10%) will be accounted for in the statistical analysis.

### Impact of the COVID-19 Pandemic

In early 2020, the COVID-19 pandemic, which first affected China followed by other APAC countries, caused widespread disruptions to trial conduct, including mandated recruitment holds, disrupted and out-of-window patient visits and follow-up, and effects on health care staffing with diversion to other roles, drug supply, and biospecimen collections.^20^ Adaptive strategies were swiftly applied such as telehealth and telephone consults, localized blood and imaging investigations, direct postage of drugs to participants, consent form amendments, and rationalized recruitment and biospecimen collection. Initially these measures were response driven and initiated by affected sites. Later, the Trial Steering Committee standardized and endorsed a set of advice, including study document amendments and preemptive processes for future similar circumstances. Overall, the greatest disruption occurred in the earlier half of 2020, with resumption of trial activity at most sites by fourth quarter of 2020. The long-term impact on the trial included prolongation of study timeline by a further 6 months, and possible missing or delayed data that may affect end point evaluation, including a likely increased proportion of participants who are lost to follow-up. The early fears of many COVID-19–related deaths were fortunately not realized, although remain a potential concern for future waves.

## DISCUSSION

The global clinical trial landscape has shifted from US- and Europe-centered activity with the greatest growth now being observed in the APAC region, which has the largest population of patients with cancer, including an increase of 138% between 2010 and 2020 in oncology clinical trials.^21^ Between 2017 and 2021, over 50% of clinical trials were conducted in the APAC region followed by the United States (29%) and Europe (17%).^22^ The unique advantages of this region include large and ethnically diverse populations, regulatory reforms facilitating easier research conduct in many countries, relative cost-efficiency, and existing and growing expertise in skilled personnel and trial infrastructure. Additionally, regulatory authorities in countries including Japan and China require locally acquired data for licensing approvals. There are also scientific and ethical motivations to conduct trials in different populations, to understand pharmcoethnogenomic implications, and to ensure participant and investigator equity and diversity, as recognized by the 2022 US Food and Drug Administration (FDA)'s draft guidance on clinical trial diversity.^23^

Despite the obvious rewards, the APAC region has over 40 economies with varying technical capabilities and health care systems. Initiating and conducting academic clinical trials with limited funding across these diverse jurisdictions, as we have experienced, is challenging and often arduous. A key factor underlying this complexity as well as the rising cost and duration of trials is heterogeneous, cumbersome, and antiquated regulatory and legal pathways, and implementation processes. This was recognized by a policy report from the Organisation for Economic Co-operation and Development Working Group on international noncommercial clinical trials^24^ and the US FDA co-founded Clinical Trials Transformation Initiative.^25^ Among the evidence-based recommendations by these groups are international coordination and harmonization of administrative requirements and adopting purpose-built rather than one-size-fits-all approaches (eg, risk-based monitoring) while maintaining high ethical and scientific standards. The European Union Clinical Trials Regulation (No 536/2014), which entered into application in January 2022, introduced a series of initiatives to streamline clinical trial conduct in Europe such as a single online registration portal and simplified safety reporting for ‘low-intervention clinical trials’, reversing the 2001 directives (2001/20/EC) that resulted in significant loss of activity in the sector, particularly for academic trials because of overly stringent regulations.^26,27^

Many opportunities exist in the APAC region to promote efficient and patient-centered oncology trials (Table 2). First, a long-term goal may be to re-engineer and simplify relevant regulations and requirements across jurisdictions to achieve reciprocal recognition and work toward harmonization. These include increasing the use of centralized ethics board reviews, standardize indemnity processes, establish best practice guidelines for biospecimen collection and sharing, and more permissive and innovative frameworks for data sharing.^28^ Globally, multiple agencies have demonstrated regulatory agility and engagement during the COVID-19 pandemic,^29^ providing cause for cautious optimism. At the regulatory approval level, collaborative Rreview between regulatory partners can substantially reduce duplications and accelerate approval of effective therapies. International initiatives have included Project Orbis led by the US FDA, including two APAC partners (Australia and Singapore)^30^ and the World Health Organization Collaborative Registration Procedures.^31^ Second, deliberate investment and commitment by governments, funding agencies, and industry to support academic collaborative trials in the form of funding and health care–related incentives are critical, particularly for low- and middle-income economies. To overcome geographical, socioeconomic, and cultural disparities, patient-centered recruitment and monitoring should be strongly encouraged, leveraging digital technologies and where possible, adopting ‘hub and spoke’ models to reach broader communities.^32^ Beyond the direct scientific and clinical gains, cross-country collaborations have the potential to benefit less experienced sites and underserved regions via training of personnel and improvement in local research infrastructure. Third, pan-APAC cooperative groups or consortia may be hugely beneficial to unify efforts, provide governance, and an avenue for advocacy with governments and industry. Such a consortium should build infrastructure for an academic clinical research unit, with support and guidance for protocol writing, grant applications, contractual services, and linkages to translational capabilities. Integration and alliance with other global clinical trial networks to facilitate exchange of ideas, data sharing, and APAC leadership should also be prioritized. The end goal for all stakeholders is to build a sustainable, fair, coherent, and inclusive clinical research ecosystem while harnessing the region's dynamism and innovation.

## References

[1] Atal I Trinquart L Porcher R et al Differential globalization of industry- and non-industry–sponsored clinical trials PLoS One 10 e0145122 2015 26658791 10.1371/journal.pone.0145122 PMC4681996

[2] Leddy L Sukumar P O’Sullivan L et al An investigation into the factors affecting investigator-initiated trial start-up in Ireland Trials 21 962 2020 33228755 10.1186/s13063-020-04893-z PMC7684941

[3] Duffett M Choong K Foster J et al High-quality randomized controlled trials in pediatric critical care: A survey of barriers and facilitators Pediatr Crit Care Med 18 405 413 2017 28328786 10.1097/PCC.0000000000001144

[4] Seruga B Sadikov A Cazap EL et al Barriers and challenges to global clinical cancer research Oncologist 19 61 67 2014 24323390 10.1634/theoncologist.2013-0290 PMC3903063

[5] Ali R Toh HC Chia WK The utility of Aspirin in dukes C and high risk dukes B colorectal cancer—The ASCOLT study: Study protocol for a randomized controlled trial Trials 12 261 2011 22168568 10.1186/1745-6215-12-261 PMC3271983

[6] Middle income | Data, 2023 https://data.worldbank.org/country/XP

[7] Sung H Ferlay J Siegel RL et al Global cancer statistics 2020: GLOBOCAN estimates of incidence and mortality worldwide for 36 cancers in 185 countries CA Cancer J Clin 71 209 249 2021 33538338 10.3322/caac.21660

[8] Guraya SY Pattern, stage, and time of recurrent colorectal cancer after curative surgery Clin Colorectal Cancer 18 e223 e228 2019 30792036 10.1016/j.clcc.2019.01.003

[9] Thierry A Corrado B Lamia MB et al Oxaliplatin, fluorouracil, and leucovorin as adjuvant treatment for colon cancer N Engl J Med 350 2343 2351 2004 15175436 10.1056/NEJMoa032709

[10] Argilés G Tabernero J Labianca R et al Localised colon cancer: ESMO Clinical Practice Guidelines for diagnosis, treatment and follow-up Ann Oncol 31 1291 1305 2020 32702383 10.1016/j.annonc.2020.06.022

[11] Rothwell PM Wilson M Elwin CE et al Long-term effect of aspirin on colorectal cancer incidence and mortality: 20-year follow-up of five randomised trials Lancet 376 1741 1750 2010 20970847 10.1016/S0140-6736(10)61543-7

[12] Fuchs C Meyerhardt JA Heseltine DL et al Influence of regular aspirin use on survival for patients with stage III colon cancer: Findings from Intergroup trial CALGB 89803 J Clin Oncol 23 2005 suppl 16; abstr 3530

[13] Chan AT Ogino S Fuchs CS Aspirin use and survival after diagnosis of colorectal cancer JAMA 302 649 658 2009 19671906 10.1001/jama.2009.1112 PMC2848289

[14] Bastiaannet E Sampieri K Dekkers OM et al Use of Aspirin postdiagnosis improves survival for colon cancer patients Br J Cancer 106 1564 1570 2012 22454078 10.1038/bjc.2012.101 PMC3341868

[15] Chia WK Ali R Toh HC Aspirin as adjuvant therapy for colorectal cancer—Reinterpreting paradigms Nat Rev Clin Oncol 9 561 570 2012 22910681 10.1038/nrclinonc.2012.137

[16] Home—ClinicalTrials.gov, 2023 https://clinicaltrials.gov/ct2/home

[17] NIPH clinical trials search, 2023 https://rctportal.niph.go.jp/en/detail?trial_id=jRCTs031180009

[18] Aspirin Trialist Collaborative Group | LUMC, 2023 https://www.lumc.nl/en/patient-care/meedoen-aan-wetenschappelijk-onderzoek/lopende-onderzoeken/aspirin-trialist-collaborative-group/

[19] Abbott L Grady C A systematic review of the empirical literature evaluating IRBs: What we know and what we still need to learn J Empir Res Hum Res Ethics 6 3 19 2011 10.1525/jer.2011.6.1.3 PMC3235475 21460582

[20] Segelov E Prenen H Day D et al Impact of the COVID-19 epidemic on a Pan-Asian academic oncology clinical trial JCO Glob Oncol 10.1200/GO.20.00072 2020 10.1200/GO.20.00072 PMC7193776 32293940

[21] Mahmood S Asia-Pacific region has seen the largest growth in oncology-related trials over the past decade. Clinical Trials Arena, 2023 https://www.clinicaltrialsarena.com/features/asia-pacific-region-has-seen-the-largest-growth-in-oncology-related-trials-over-the-past-decade/

[22] Evolution of clinical trials in the Asia Pacific region compared to the US and the EU5. Novotech CRO, 2023 https://novotech-cro.com/whitepapers/evolution-clinical-trials-asia-pacific-region-compared-us-and-eu5

[23] Diversity plans to improve enrollment of Participa.pdf, 2022 https://www.fda.gov/media/157635/download

[24] Facilitating international cooperation in non-commercial clinical trials, 2022 https://www.oecd.org/sti/inno/49344626.pdf

[25] Tenaerts P Madre L Landray M A decade of the clinical trials transformation initiative: What have we accomplished? What have we learned? Clin Trials 15 5 12 2018 suppl 1 29452517 10.1177/1740774518755053

[26] Dittrich C Negrouk A Casali PG An ESMO-EORTC position paper on the EU clinical trials regulation and EMA’s transparency policy: Making European research more competitive again Ann Oncol 26 829 832 2015 25802239 10.1093/annonc/mdv154

[27] Regulation (EU) no 536/2014 of the European Parliament and of the Council of 16 April 2014 on clinical trials on medicinal products for human use, and repealing directive 2001/20/EC text with EEA relevance 158 2014 http://data.europa.eu/eli/reg/2014/536/oj/eng

[28] Bertagnolli MM Sartor O Chabner BA et al Advantages of a truly open-access data-sharing model N Engl J Med 376 1178 1181 2017 28328337 10.1056/NEJMsb1702054

[29] Li BT Daly B Gospodarowicz M et al Reimagining patient-centric cancer clinical trials: A multi-stakeholder international coalition Nat Med 28 620 626 2022 35440725 10.1038/s41591-022-01775-6

[30] Commissioner O of the Project Orbis. FDA, 2023 https://www.fda.gov/about-fda/oncology-center-excellence/project-orbis

[31] Vaz A Roldão Santos M Gwaza L et al WHO collaborative registration procedure using stringent regulatory authorities’ medicine evaluation: Reliance in action? Expert Rev Clin Pharmacol 15 11 17 2022 35130803 10.1080/17512433.2022.2037419

[32] Switzer JA Demaerschalk BM Xie J et al Cost-effectiveness of hub-and-spoke telestroke networks for the management of acute ischemic stroke from the hospitals’ perspectives Circ Cardiovasc Qual Outcomes 6 18 26 2013 23212458 10.1161/CIRCOUTCOMES.112.967125

